# Impressions in Orthodontia

**Published:** 1904-10-15

**Authors:** B. Abell

**Affiliations:** Albion, Mich.


### IMPRESSIONS IN ORTHODONTIA.

BY B. ABELL, D.D.S., ALBION, MICH.

Read before the Michigan Dental Association, June 29th, 1904.

Sometime ago the call came to me from Dr. Hoff for a paper on the above subject, and I find my twelve years absence from his rule that I knew in the university has not lessened my fear of disobeying him, consequently I am here to-day with the subject assigned me; a subject with which I fear I shall bore you, not because it is unworthy your attention but chiefly because it is somewhat outside the sphere of the regular practice of dentistry.

At the beginning, I want to lay no claim to originality in what I shall present, but say the method I use is entirely Dr. Angle's, to whom is due all credit for its inception, so far as I know.

In taking of impressions in orthodontia, the utmost care is essential. All deposits should be removed and the teeth well polished. The next essential is a suitable tray, and those designed by Dr. Angle seem to fill the bill satisfactorily.

They should be kept perfectly smooth and polished that they may easily separate from the impression. One should be selected large enough to admit of a good body of plaster all around buccal and labial regions. The best plaster I've found so far is French's impression plaster. For an upper impression, mixed to the proper consistency, it should be arranged along the deep portions of the tray and a considerable quantity well out on the handle, leaving the palatine portion nearly bare, as enough will flow over that to get a good impression. Inserting the tray in the mouth it should be allowed to rest on the occlusal surfaces of the lower teeth. Then with the index finger of the right hand that portion of plaster left on the handle should be wiped up under the lip and into the buccal spaces, the tray should then be brought up till the occlusal points of the cusps of the teeth are very close to the bottom of the tray. This gives a weak line along the buccal cusps where the plaster may be easily fractured for removal. The cheeks and lips should be gently manipulated to expel air when pressing the impression material home, but not drawn down to cause a thinning of the plaster above the tray.

After the plaster is well set, the tray should be removed, and the plaster impression left in the mouth. All superflous particles should be removed with large, loose pledgets of cotton. Next a groove should be cut parallel to the long axes of the cuspids, and the front section pried out with a suitable knife. The buccal and palatine portions are then easily removed and all the pieces laid on a clean blotter to dry. The small pieces should be placed near where they came away from the larger ones.

The taking of the lower varies from the upper only in that the buccal spaces are filled with plaster before the tray is inserted. The lower tray is held down by the second joints of the index fingers while the tips may be used to work the plaster well down below edge of tray. On removing the lower, a good sized piece will usually be found under the tongue and between the wings of the tray. This should be removed before tray is, and saved to brace the impression when united.

After the impression is thoroughly dry the small pieces are united to the larger ones by a cement made of celluloid, cut with equal parts ether and alcohol. The larger ones are united with wax outside the tray. If care is exercised in trying the pieces together but once, the fine serrations will not be rubbed off and the union will be scarcely perceptible.

To obtain a smooth model, the impression is first coated with orange shellac varnish. It is desirable that this coat should penetrate the impression deeply for reasons that will appear later. When dry, another coat of thin shellac is applied and made to dry quickly to give surface. This coat is followed by a coat of sandarac varnish, omitting to cover the stippled portion of the gums with both second and third coats mentioned. The impression is now ready to pour, which is done without wetting the impression, using a good sized camel's hair brush to sweep the plaster into the pits.

The impression is removed by first carving almost to the coronal surfaces of the teeth. The danger line being the brown color from the first coat of shellac that has penetrated the impression. A groove is next cut down to color on a line parallel with the gingivea and cross cuts made leaving the impression in blocks that are easily pried off with a suitable knife.

The carving of the model next engages our attention and for those who want artistic effects there are certain rules that we follow more or less closely. We aim as nearly as possible to have the sides represent the malar bones, this indicating approximately any deviation lateral to normal the teeth may occupy. The height is merely a matter of taste, and is determined by one's sense of "balance."

I've been asked to give the *modus operandi* of impression taking, and the telling of it sounds dry indeed, but I assure you the doing of it is no dry operation, as any of the patients will tell you, and unless one is careful he will be reminded of a verse in the "Charge of the Light Brigade," only it will be "*Plaster* to right of them, *plaster* to left of them," etc., and the office girl be overheard to breathe *blessings* upon you as she sweeps up the debris.

Plaster impressions and models showing the operation in different stages were exhibited, making the method described very clear.

DISCUSSION.

Dr. E. C. Moore: Said he could see no great advantage to be gained by the use of French's plaster, as it was very fine, and did not set hard like the coarser plasters. He could see no great advantage in breaking the impression, as in many cases it was practically impossible to reassemble the pieces accurately.

Dr. Abell explained that the fine plaster set quicker and gave a smoother surface to the case because it was so fine. The impression in almost all cases had to be broken to be able to remove them from the mouth, as the teeth were so irregular that they would not permit of the withdrawal without fracture. It was also a valuable fact that the fine plaster did not set so hard that it might not be removed without causing much pain. If reasonable care and skill are used the pieces can be put together accurately enough. The impression should be well dried before they are varnished.

Dr. I. Douglas: For making celluloid cement for mending casts, dissolve the celluloid in acetone, as it is better than ether and alcohol. This cement may be profitably used in place of collodion on wounds, etc., like court plaster, to protect from the air.

Dr. Sweetnam: I can not too strongly urge the necessity of making good models and also of preserving these for future reference. I recently completed a very difficult case of regulating, but neglected to secure and keep the good set of models at the start. I would gladly give the fee I received for a good set of plaster models of this case before the treatment was begun. The models have greater value for diagnosing the case and keeping records as the treatment progresses.

Dr. Hoff: Dr. Abell apologized for bringing up this subject at this time. But I want to say that it is a subject that sorely needs consideration. I am in a position to notice the carelessness of the average practitioner in taking impressions of cases of irregularities of the teeth. I frequently get models for advice as to treatment. And I can say that out of the many cases sent me I do not get one in a dozen that I can make an accurate diagnosis from. Often a mere wax bite or a carelessly taken compound impression provides a model—generally of one jaw only—which is utterly valueless for any thing more than a guess opinion. We can't get these models too perfect to secure the best results. Every tooth, and even the rugal on the palatal surface as well as the eminence and depression of the process, should be sharply and definitely outlined, and the cusps in perfect condition are absolutely necessary, if we are to eliminate guess work, and secure scientific results in treatment. The models should *always* be taken with plaster and made as carefully as though they were to adorn the niches of the most famous art galleries. I regret that Dr. Abell has not dwelt more on the details of this operation which has become simple to him, but which I am sure the majority of us look upon as a matter of slight consequence, when it is one of very great importance. I believe that if we were to spend as much care and time in obtaining models for prosthodontia as specialists do for orthodontia that we should have better success in fitting artificial dentures.

Dr. Watson: I let my impression dry for at least three hours, before attempting to put it together, as the cement will not adhere to the wet plaster. It requires lots of skill and tact to secure good models from some of the cases which present themselves, and we often have to modify methods to secure suitable models. A good set of models is the first step in attempting to treat any case of mal-occlusion. The models should be in pairs, even should the mal-occlusion be confined to a single tooth in one jaw. Perfect models are not necessary for the use of constructing appliances. We don't make appliances now, we buy them for the same reason that dentists buy burs rather than to undertake their manufacture. Unsightly mal-occlusions bring us most of our patients probably. It is however true that pathological conditions of the gums send us many patients. In these conditions it is practically essential that the best models obtainable should be secured. We can not study a case thoroughly from the mouth only. Models give us cues to the causes as well as methods of treatment, and we can not err seriously if we have a good model to study at leisure.

Dr. Abell: In separating the impression from the model the character of the teeth should be remembered, and the impression carved into suitable sections which can be removed with the point of a good pen-knife. If defects occur paint the models with thinly-mixed plaster and let it harden and then carve to the right form and occlusion. An entire tooth may be built up in this way. French chalk gives a smooth surface to models, but many do not use it.

